# Diagnostic performance of ^18^F-FDG PET/CT using point spread function reconstruction on initial staging of rectal cancer: a comparison study with conventional PET/CT and pelvic MRI

**DOI:** 10.1186/s40644-018-0137-9

**Published:** 2018-01-30

**Authors:** Masatoshi Hotta, Ryogo Minamimoto, Hideaki Yano, Yoshimasa Gohda, Yasutaka Shuno

**Affiliations:** 1 Division of Nuclear Medicine, Department of Radiology, National Center for Global Health and Medicine, 1-21-1, Shinjuku-ku, Toyama, Tokyo 162-8655 Japan; 2 Department of Surgery, National Center for Global Health and Medicine, 1-21-1, Shinjuku-ku, Toyama, Tokyo 162-8655 Japan

**Keywords:** Point spread function (PSF), 18F-FDG pet/ct, Pelvic MRI, Rectal cancer, Staging

## Abstract

**Background:**

Accurate staging is crucial for treatment selection and prognosis prediction in patients with rectal cancer. Point spread function (PSF) reconstruction can improve spatial resolution and signal-to-noise ratio of PET imaging. The aim of this study was to evaluate the effectiveness of ^18^F-FDG PET/CT with PSF reconstruction for initial staging in rectal cancer compared with conventional PET/CT and pelvic MRI.

**Methods:**

A total of 59 patients with rectal cancer underwent preoperative ^18^F-FDG PET/CT and pelvic MRI. The maximum standardized uptake value (SUVmax) and lesion to background (L/B) ratio of possible metastatic lymph nodes, and metabolic tumor volumes (MTVs) of primary tumors were calculated. For N and T (T1-2 vs T3-4) staging, sensitivities, specificities, positive predictive values, negative predictive values, and accuracies were compared between conventional PET/CT [reconstructed with ordered subset expectation maximization (OSEM)], PSF-PET/CT (reconstructed with OSEM+PSF), and pelvic MRI. Histopathologic analysis was the reference standard.

**Results:**

For N staging, PSF-PET/CT provided higher sensitivity (78.6%) than conventional PET/CT (64.3%), and pelvic MRI (57.1%), and all techniques showed high specificity (PSF-PET: 95.4%, conventional PET: 96.7%, pelvic MRI: 93.5%). SUVmax and L/B ratio were significantly higher in PSF-PET/CT than conventional-PET/CT (*p* < 0.001). The accuracy for T staging in PSF-PET/CT (69.4%) was not significantly different to conventional PET/CT (73.5%) and pelvic MRI (73.5%). MTVs of PSF and conventional PET showed a significant difference among T stages (p < 0.001), with higher values in advanced stages. In M staging, both PSF and conventional PET/CT diagnosed all distant metastases correctly.

**Conclusions:**

PSF-PET/CT produced images with higher lesion-to-background contrast than conventional PET/CT, which allowed improved detection of lymph node metastasis without compromising specificity, and showed comparable diagnostic value to MRI in local staging. PSF-PET/CT is likely to have a great value for initial staging in rectal cancer.

## Background

Incidence of rectal cancer is relatively high and one of the major causes of cancer-related mortality worldwide [1]. Accurate initial staging is important for determining prognosis and treatment options in patients with rectal cancer [2–4]. While CT and MRI are commonly used for initial staging of rectal cancer, their diagnostic performance for N staging is not entirely satisfactory [5, 6]. Small lymph node metastases are common in rectal cancer, and these can be difficult to diagnose by CT and MRI, frequently resulting in false negatives which can lead to incorrect management decisions. In contrast, ^18^F-FDG PET/CT has been shown to have high specificity for the diagnosis of lymph node (LN) metastasis in rectal cancer [7–10], as in addition to size criteria it evaluates glucose metabolism.

Recently, the point spread function (PSF) reconstruction technique has become commercially available for PET imaging [11, 12]. PSF reconstruction corrects photon mis-positioning (parallax effect) while gamma rays pass in the scintillation detectors at both non-oblique and oblique angles. This algorithm can improve the spatial resolution and signal-to-noise ratio of PET images [13, 14], leading to higher detection rates for small lesions. The latest PET scanners generally equipped with PSF reconstruction, which can be used only by changing its reconstruction algorithm, without additional image acquisition. ^18^F-FDG PET/CT using PSF reconstruction has already been reported to improve the sensitivity of nodal staging for malignancies such as lung or breast cancer [15–17]. However, the utility of PSF for in rectal cancer has not been adequately clarified.

The aim of this study was to evaluate the effectiveness of ^18^F-FDG PET/CT using PSF reconstruction for the initial staging in patients with rectal cancer, and compare it to both pelvic MRI and ^18^F-FDG PET/CT using ordered subset expectation maximization (OSEM) reconstruction.

## Methods

### Patients

This retrospective study was approved by the Institutional Review Board of our hospital, and the need to obtain informed consent was waived.We included histologically proven rectal cancer patients who underwent ^18^F-FDG PET/CT and pelvic MRI for their initial staging from November 2011 to August 2016. Exclusion criteria for this study were: patients with uncontrolled diabetes, and patients who were not indicated for surgery and therefore underwent preoperative radiation therapy or chemotherapy after PET evaluation.

### Surgical protocol

The surgical protocol, including dissection area, followed the Japanese Society for Cancer of the Colon and Rectum (JSCCR) guidelines [18]. Patients with locally advanced lower rectal cancer had undergone pelvic sidewall dissection because of a greater probability of positive lateral lymph nodes [19]. Patients with upper rectal cancer showing possible lateral lymph node metastasis on preoperative imaging such as MRI, PET/CT, or enhanced-CT had undergone pelvic sidewall dissection, regardless of tumor location or T stage.

### PET/CT examination

^18^F-FDG was synthesized with an in-house cyclotron and automated synthesis system (F200; Sumitomo Heavy Industries, Shinagawa, Tokyo, Japan). PET/CT images were acquired 60 min after an intravenous injection of ^18^F-FDG, fixed at 5.0 MBq/kg. Patients were instructed to urinate before scanning to reduce tracer accumulation in the bladder. All PET/CT images were obtained using a Discovery PET/CT 600 (GE Healthcare, Pewaukee, WI) with a multi-detector-row CT component (16 detectors). Scanning covered an area from the head to the mid-thigh. Low dose CT with shallow breathing was performed first and used for attenuation correction and image fusion. CT acquisition was performed with 120 kVp using an auto exposure control system, beam pitch of 0.938, slice thickness of 3.75 mm. Emission images were acquired in three-dimensional mode for 2.5 min per bed position. The 3D-OSEM reconstruction method (VUE point HD; GE Healthcare) was used both for (a) conventional PET (16 subsets; 3 iterations), and (b) PSF-PET [(16 subsets; 5 iterations) + PSF algorithm (Sharp IR, GE Healthcare)]. For both reconstructions, the matrix size was 192 × 192, resulting in a 3.65 × 3.65 × 3.65 mm voxel size, and a 4 mm Gaussian filter was used.

### MRI examination

MRI was performed with either a 1.5-T scanner (Avanto; Siemens Medical Systems, Erlangen, Germany) or a 3-T scanner (Verio; Siemens Medical Systems, Erlangen, Germany). The allocation of patients to both MRI scanners were performed randomly.

The following are imaging parameters for the acquired sequences, for the 1.5-T and the 3-T scanners, respectively. Axial T1-weighted images: repetition time (TR)/echo time (TE), 666/11 ms, matrix size, 192 × 320, slice thickness, 6 mm, intersection gap, 1.5 mm, number of excitations (NEX), 2, and field of view, 25 × 25 cm; TR/TE, 550/11 ms, matrix size, 256 × 320, slice thickness, 6 mm; intersection gap, 1.5 mm, NEX, 1, and field of view, 24 × 24 cm. Oblique (perpendicular to the tumoral axis) high resolution T2-weighted images: TR/TE, 5850/91 ms, matrix size 230 × 256, slice thickness, 3 mm, intersection gap, 0 mm, NEX, 1, and field of view, 20 × 20 cm; TR/TE, 5000/100 ms; matrix size 256 × 256, slice thickness, 3 mm, no gap, NEX, 1, and field of view, 18 × 18 cm. Axial diffusion-weighted images (DWI) were acquired using free breathing, single-shot acquisition, short tau inversion recovery (STIR)–echo planar imaging (EPI) sequence: TR/TE, 7600/75 ms, matrix size 79 × 128, slice thickness, 5 mm, no gap, NEX, 6, field of view, 36 × 36 cm, and b-value, 1000 s/mm^2^; TR/TE, 9600/72 ms, matrix size 128 × 128, slice thickness, 5 mm, no gap, NEX, 5, field of view, 35 × 35 cm, and 1000 s/mm^2^.

### Image analysis

All PET/CT examinations were evaluated by consensus of two board-certified nuclear medicine physicians blind to clinical and pathological information. PSF and conventional PET/CT images were accessed independently. For N staging, lymph nodes that showed abnormal uptake compared to surrounding tissue were considered positive, regardless of size. For semi-quantitative analysis, a region of interest (ROI) was contoured over possible metastatic lymph nodes and maximum standard uptake values (SUVmax) were calculated. To measure background uptake, a circular ROI of 10 mm diameter was placed on the ascending aorta according to the CT image of the PET/CT. For both conventional PET/CT and PSF-PET/CT, a lesion-to-background (L/B) ratio was calculated from the values of the lymph node and background uptakes.

MRI images were anonymized and evaluated by consensus of two board-certified diagnostic radiologists. For N staging, lymph nodes greater than 8 mm in diameter, or that showed either irregular contour or mixed signal intensity in T2-weighted images were considered as metastasis [20, 21]. DWI was used for aiding in the detection of lymph nodes only, as DWI is not considered reliable for differentiating between benign and malignant lymph nodes with non-metastatic lymph nodes showing restricted diffusion [22, 23]. In both PET/CT and MRI, lymph nodes were evaluated on a per region basis: mesorectal, superior rectal, inferior mesenteric, internal iliac, and obturator. This anatomical grouping was based on the modified American Joint Committee on Cancer (AJCC) staging system [24], reported by Kim et al. [25]. For T staging, tumors that showed extended ^18^F-FDG uptake or soft tissue density to surrounding mesorectal fat were considered as over T3 (T3-4). According to the AJCC staging system, tumors that invade perirectal tissue are classified as T3, and those that directly invade other organs are classified as T4. Metabolic tumor volumes (MTVs) were measured from ^18^F-FDG PET images using the PET Edge tool (MIM software, Cleveland, OH), which creates boundary contours automatically detecting the steepest drop-off in SUV according to a gradient-based technique [26]. In MRI, tumors that showed invasion of mesorectal fat on T2-weighted images were considered as over T3 stage (T3-4).

These imaging findings for T and N staging were compared with histopathological analysis of the primary tumor and harvested lymph nodes. In addition, M staging was assessed in PET/CT and MRI, with the reference standard for metastasis set from the patient’s clinical course and following scans including FDG PET/CT and contrast-enhanced CT.

### Statistical analysis

Data are expressed as mean ± SD. Sensitivity, specificity, positive predictive value (PPV), negative predictive value (NPV), and accuracy values for T staging (T1-2 vs T3-4) and N staging were calculated for conventional PET/CT, PSF-PET/CT, and MRI (these values are expressed as means with 95% confidence intervals [CI]). The McNemar chi-square test was used to compare the sensitivity and specificity between PET/CT, PSF-PET/CT, and MRI. Wilcoxon signed-rank test was performed to compare SUVmax and L/B ratio of positive lymph nodes, and MTVs of the primary lesion between PSF and conventional PET. The relationship of SUVmax and L/B ratio between PSF and conventional PET was assessed by linear regression analysis. Kruskal-Wallis test was used for comparing the difference of MTVs among T stages, and receiver operating curve (ROC) analysis was performed to evaluate the diagnostic performance of MTVs to distinguish the T3-4 from T1-2 stages. Two-tailed *p* values < 0.05 were considered significant.

## Results

### Clinical data

Fifty-nine patients met the study inclusion criteria and, of these, 10 patients underwent radiation therapy or chemotherapy. No patient showed serum glucose concentrations > 150 mg/dL prior to ^18^F-FDG administration. A final number of 49 patients were included in the analysis (Table 1). The median interval between PET/CT and MRI, between surgery and PET/CT, and between surgery and MRI were 4 (interquartile range: 2-8), 11 (7-23), and 17 (9-27) days, respectively. Twenty-one patients underwent MRI with the 3-T scanner, and 28 patients with the 1.5-T scanner.

**Table 1:** Patients demographics

No. patients	49
Sex	M 34, F 15
Mean Age years (standard deviation)	66.8 (12.9)
Histological diagnosis	
Well differentiated adenocarcinoma	17
Moderately differentiated adenocarcinoma	29
Poorly differentiated adenocarcinoma	1
Mucinous adenocarcinoma	2
Pathological Stage (UICC)	
I	13
II	15
III	16
IV	5

*M* male, *F* female, *UICC* Union for International Cancer Control

### Diagnostic performance for N staging

**Fig. 1:**
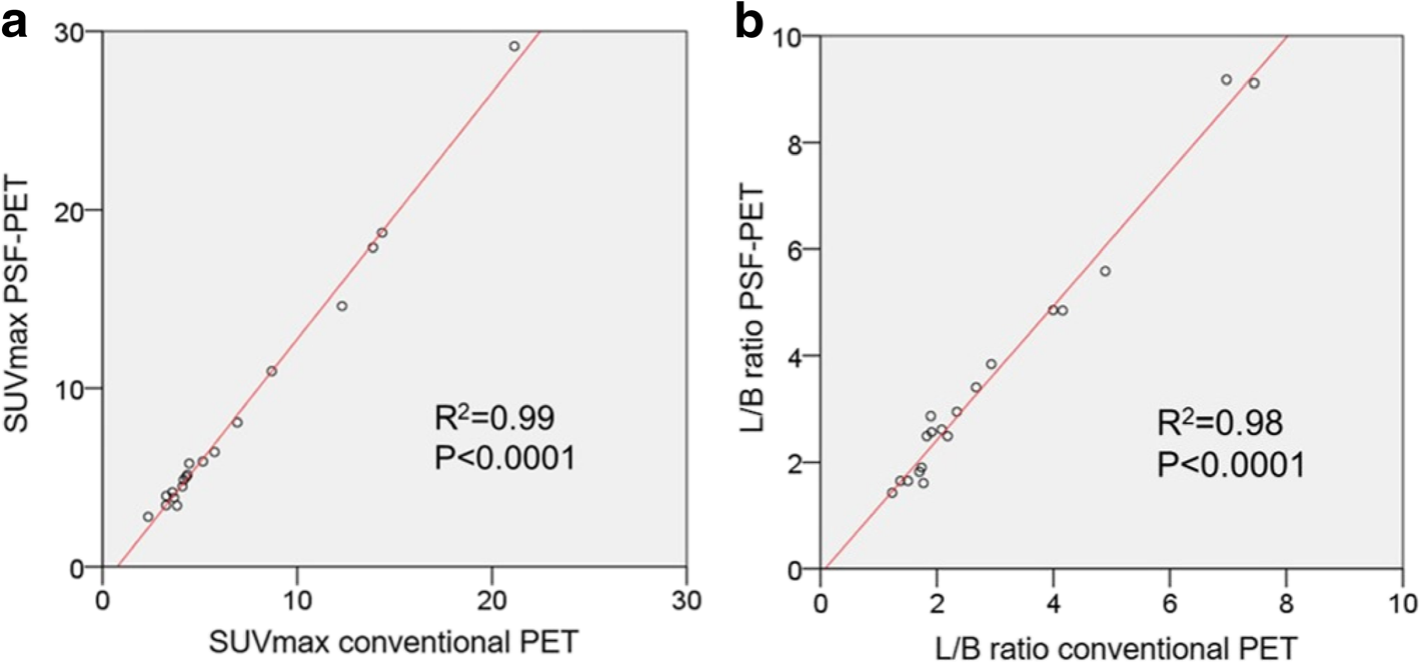
Relationship between quantitative values obtained from conventional PET and point spread function (PSF) -PET, evaluated using linear regression analysis for maximum standardized uptake value (SUVmax) (**a**), and lesion to background (L/B) ratio (**b**)

**Fig. 2:**
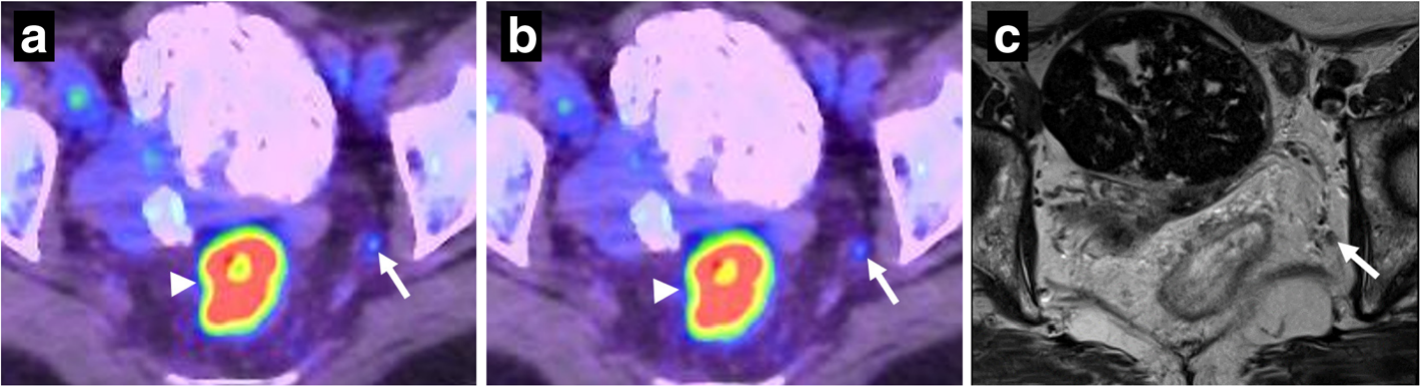
A 83-year-old woman with rectal cancer. The rectal cancer (arrowhead) can be seen from ^18^F-FDG uptake on both PSF-PET/CT (**a**) and conventional PET/CT (**b**) [images are scaled to the same maximum value]. A obturator lymph node (arrow) showed as ^18^F-FDG avid compared to the surrounding tissue on PSF-PET/CT, and therefore considered a positive result. This lymph node was obscure on conventional PET/CT, and thus regarded as negative. This lymph node was 5 mm in diameter, and did not show mixed signal intensity nor irregular contour on the high resolution T2-weighted image (**c**), and therefore considered negative on MRI also. Subsequently, this lymph node was pathologically confirmed as containing metastasis

A total of 1200 LNs were resected by surgery, and 104 (8.7%) LNs were pathologically proven metastasis. On a per patient basis, 18 (36.7%) patients showed lymph nodal involvement. On a per region basis, the prevalence of lymph node metastasis was 15.5% (28/181) [mesorectal (*n* = 18/49), superior rectal (*n* = 3/49), inferior mesenteric (*n* = 2/45), internal iliac (n = 3/20), obturator (n = 2/18)]. The diagnostic performances for N staging on a per region basis are shown in Table 2. The sensitivity of PSF-PET/CT (78.6%) was higher than that of either conventional PET/CT (64.3%) or pelvic MRI (57.1%) but the differences were not statistically significant (PSF-PET vs conventional PET, *p* = 0.13; PSF-PET vs MRI, *p* = 0.07). PSF-PET/CT showed positive uptake for all the true positive lesions (*n* = 17) on conventional PET/CT. Metastasis in normal size lymph nodes was seen in 9/28 (32.1%) lesions. Of these, 4/9 (44.4%) and 1/9 (11.1%) were detected on PSF-PET/CT and conventional PET/CT, respectively. No significant differences were observed for specificity, PPV, NPV, or accuracy between the three methods. The average SUVmax and L/B ratios for visually positive regions are shown in Table 3. The increased percentages of SUVmax and L/B ratios by PSF reconstruction were 17% and 21%, respectively. The L/B ratio was significantly higher with PSF-PET/CT than with conventional PET/CT (*P* < 0.001). Linear regression analyses are shown in Fig. 1. An excellent correlation was found between quantitative measurements extracted from conventional PET/CT and PSF-PET/CT for SUVmax, and L/B ratios, with an r^2^ value greater than 0.9. Similar results were found for SUVmax ratios. Figure 2 shows a representative case, comparing PSF-PET/CT, conventional PET/CT, and MRI.

**Table 2:** Diagnostic performance of conventional PET/CT, PSF PET/CT, and pelvic MRI for nodal staging in patients with rectal cancer

	Conventional PET/CT	PSF-PET/CT	Pelvic MRI
Sensitivity, %	64.3 (0.51 to 0.73)	78.6 (0.65 to 0.88)	57.1 (0.42 to 0.69)
Specificity, %	96.7 (0.94 to 0.98)	95.4 (0.93 to 0.97)	93.5 (0.91 to 0.96)
Accuracy, %	91.7 (0.88 to 0.95)	92.8 (0.89 to 0.96)	87.8 (0.83 to 0.92)
PPV, %	78.3 (0.62 to 0.89)	75.9 (0.63 to 0.85)	61.5 (0.46 to 0.75)
NPV, %	93.7 (0.91 to 0.95)	96.1 (0.94 to 0.98)	92.3 (0.90 to 0.95)
Positive LR	19.6 (8.80 to 45.60)	17.2 (9.10 to 30.50)	8.7 (4.60 to 16.20)
Negative LR	0.37 (0.27 to 0.52)	0.23 (0.13 to 0.38)	0.46 (0.32 to 0.63)

The numbers in parentheses represent the 95% confidence interval

*LR* likelihood ratio, *NPV* negative predictive value, *PPV* positive predictive value, *PSF* point spread function

**Table 3:** Quantitative values of conventional PET/CT and PSF-PET/CT

	Conventional PET/CT	PSF-PET/CT	P value*
SUVmax	6.8 (5.0)	8.4 (7.0)	< 0.001
Background	2.3 (0.3)	2.2 (0.5)	0.32
L/B ratio	2.9 (1.8)	3.5 (2.3)	< 0.001

The numbers in parentheses represent the standard deviation. *Wilcoxon signed-rank test

*L/B* lesion to background, *PSF* point spread function, *SUV* standardized uptake value

### Diagnostic performance for T and M staging

**Fig. 3:**
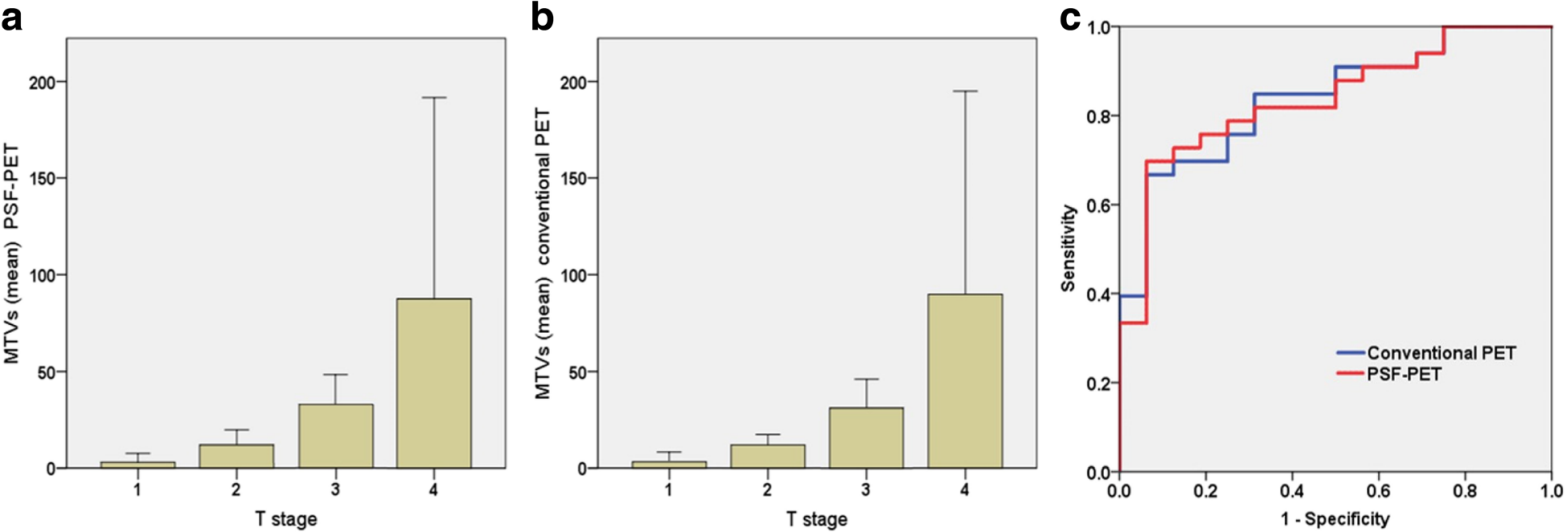
Mean metabolic tumor volumes (MTVs) of each T stage for point spread function (PSF)-PET (**a**), and for conventional PET (**b**). Error bars show 95% confidence intervals. Mean MTVs of PSF and conventional PET were 3.0 ± 4.4, 3.2 ± 4.8 for T1, 12.1 ± 10.7, 11.9 ± 7.6 for T2, 32.9 ± 37.6, 31.1 ± 36.1 for T3, and 87.6 ± 124.4, 89.8 ± 125.8 for T4, respectively. Receiver operating characteristic curves (ROC) analysis (**c**) for discriminating T3-4 stage using MTVs of PSF and conventional PET

Pathological T staging of patients was as follows: T1 (*n* = 6, 12.2%), T2 (*n* = 10, 20.4%), T3 (*n* = 25, 51.0%), and T4 (*n* = 8, 16.3%). The diagnostic performances for visual differentiation of the T3-4 stage between conventional PET/CT, PSF-PET/CT, and MRI are presented on Table 4. Pelvic MRI showed higher sensitivity (70.0%) than that of PSF-PET/CT (57.6%) or conventional PET/CT (63.6%), but the differences were not statistically significant (pelvic MRI vs PSF-PET/CT, *p* = 0.29; pelvic MRI vs conventional PET/CT, *p* = 0.72). Figure 3 (a and b) shows MTVs of PSF-PET and conventional PET respectively, both showing significant differences (*p* < 0.001) among T stages. There were no significant differences for mean MTVs between PSF and conventional PET (PSF-PET: 33.9 ± 60.7, conventional PET33.7 ± 60.9, *p* = 0.57). Figure 3c shows the ROC analysis of PSF and conventional PET for determining T3-4. The AUC for PSF-PET was 0.837 and 0.839 for conventional PET, which showed no statistical difference (*p* = 0.91). When a cut-off value of MTVs was set at 18.1 mL in conventional PET, a sensitivity of 93.8% and specificity of 69.7% were obtained. This diagnostic value was as high as that of the MTVs of PSF-PET.

**Table 4:** Diagnostic performance of conventional PET/CT, PSF PET/CT, and pelvic MRI for differentiating T3-4 stage from T1-2 stage in patients with rectal cancer

	Conventional PET/CT	PSF-PET/CT	Pelvic MRI
Sensitivity, %	63.6 (0.45 to 0.80)	57.6 (0.39 to 0.74)	70.0 (0.51 to 0.84)
Specificity, %	93.8 (0.70 to 0.99)	93.8 (0.70 to 0.99)	81.2 (0.54 to 0.96)
Accuracy, %	73.5 (0.59 to 0.85)	69.4 (0.55 to 0.82)	73.5 (0.59 to 0.85)
PPV, %	95.5 (0.77 to 0.99)	95.0 (0.75 to 0.99)	88.8 (0.70 to 0.98)
NPV, %	55.6 (0.35 to 0.74)	51.7 (0.33 to 0.71)	56.5 (0.59 to 0.85)
Positive LR	10.2 (1.50 to 69.10)	9.2 (1.30 to 62.80)	3.7 (1.30 to 10.60)
Negative LR	0.38 (0.24 to 0.62)	0.45 (0.30 to 0.69)	0.37 (0.21 to 0.66)

The numbers in parentheses represent the 95% confidence interval

*LR* likelihood ratio, *NPV* negative predictive value, *PPV* positive predictive value, *PSF* point spread function

With regards to M staging, both PSF PET/CT and conventional PET/CT detected all distant metastasis [5/49 (10.2%) patients; 2 cases for lung, 1 case for liver, 1 case for para-aortic LNs, and 1 case for lung, liver, and bone metastasis], whereas pelvic MRI was not able to diagnose distant metastases because of its scanning range limitations.

## Discussion

The purpose of this study was to investigate whether PSF-PET/CT improves diagnostic performance for initial staging of patients with rectal cancer, as compared to conventional PET/CT and pelvic MRI. For nodal staging, PSF-PET/CT provided higher sensitivity, without decreasing specificity, than conventional PET/CT and pelvic MRI. SUVmax and L/B ratio of PSF reconstruction were significantly higher than those of conventional OSEM reconstruction. For T staging, both PSF and conventional PET/CT provided similar diagnostic performance to pelvic MRI, in terms of discriminating stages T3-4 from T1-2.

When compared to conventional PET/CT for lymph node staging, PSF-PET/CT provided higher sensitivity than PET/CT. The increased L/B ratio can lead to the higher detectability of lymph node metastasis. This result was mostly an accordance with previous reports that examined PSF reconstruction for evaluating lymph node metastasis in malignancies including lung cancer [15, 16], breast cancer [17], and colorectal cancer [27]. However, the sensitivity improvement was relatively smaller than that in the lung and breast cancer studies. A possible explanation for this result is the use of a different PSF reconstruction algorithm. We used the Sharp IR (GE healthcare) algorithm in this study, whilst the True X (Siemens Medical Systems, Erlangen, Germany) algorithm was used in the other two studies. While both algorithm increase SUVmax and L/B ratio, True X tends to overestimate SUVmax, especially in larger lesions, when compared to Sharp IR [28]. Indeed, the increased percentages of SUVmax and L/B ratio by PSF reconstruction were 17 and 21% for our study; and 48-66% for the lung and 27-67% for the breast studies using TrueX. This difference possibly influenced the detection of lymph node metastasis. Another possible reason for the differences in sensitivity between rectal and breast cancers could be anatomical features related to ^18^F-FDG uptake. In the pelvis, physiological ^18^F-FDG uptake in the small intestine, colon and bladder causes difficulties for distinguishing abnormal uptake adjacent to these organs [29]. In this study, all patients were instructed to urinate before scanning to reduce tracer accumulation in the bladder, but controlling physiological accumulation in the small intestine and colon remains challenging. In addition, various artifacts related to hip prosthesis, which are occasionally seen particularly in elderly patients, may lower small lesions detection [30, 31]. In such cases, PET/CT image quality may be improved with the use of metal artifact reduction algorithms [32].

In this study, the detection of small lymph node metastases using PSF-PET/CT was superior to that using conventional PET/CT and MRI. In rectal cancers, almost 60% of the lymph nodes involved are smaller than 5 mm in diameter, which is a major limitation for nodal staging using the size criteria alone [20]. Therefore, as in this study, MRI has shown to be limited for detection of metastasis in normal size lymph nodes, and a relative low specificity is an issue even if specificity can be improved by combining it with characteristic MRI imaging techniques such as DWI [10]. PSF reconstruction is known to increase apparent SUV compared to OSEM, especially in case of small lesions [33]. SUVmax calculated from PSF-PET/CT itself is known to be unreliable and is not recommended in assessing treatment response or multicenter trials [28, 34]. However, it can be very useful for visually detecting small lymph node metastases, as evidenced by the higher L/B ratios in PSF-PET/CT than in conventional PET/CT seen in this study. Small lymph node commonly contains metastasis, not only in rectal cancers cases, but in other malignancies such as esophageal, gastric and uterine cervical cancers [35–37], with consequent lower lymph node metastasis detection rates. PSF PET/CT, which improves spatial resolution and small lesion detection, may also enhance the sensitivity of PET/CT for lymph node metastasis in such cancers.

The diagnostic ability of PSF and conventional PET/CT for T staging [T1-2 vs T3-4] was as high as that of MRI. It is important to distinguish T3-4 from T1-2 stages, as the former are considered advanced stages for which neoadjuvant therapy can be the first treatment option [38]. Higher MTVs were associated with more advanced T stages. Buijsen et al. [39] has reported that ^18^F-FDG PET/CT based contours show the best correlation with the tumor dimension of surgically resected specimens in rectal cancer when compared to CT and MRI. This indicates that MTVs can correlate well with the actual tumor volume, which may explain why MTVs yield high diagnostic performance for T staging. With regard to differences between PSF and conventional PET, there was no significant difference of MTVs for T staging. Previous reports described that MTVs calculated from PSF were smaller than that of conventional (OSEM reconstructed) PET, but this was not the case in the present study. Differences in PSF reconstruction algorithms and auto-segmentation software may possibly affect volume calculation [40, 41].

In this study, all distant metastasis including lung, liver, and bone were detected in PET/CT with or without PSF reconstruction. PET/CT reportedly enables accurate diagnosis for not only intrahepatic metastasis, but extrahepatic metastasis in colorectal cancer [42–45]. In this context, it would be advisable to perform ^18^F-FDG PET/CT for rectal cancer staging, particularly in advanced cases. Not only for M staging, this study has shown that PET/CT (particularly PSF-PET/CT) can provide high diagnostic performance for N staging too, and that it has a diagnostic value comparable to MRI for T staging. Collectively, PSF PET/CT has potential to become a *one-stop shop* imaging solution for initial staging in rectal cancer.

There are limitations to this study. First, it was a single center study with a relatively small population and our findings need to be confirmed in a larger series. Second, highly advanced patients who were not indicated for operation and required neoadjuvant radiotherapy or chemotherapy were excluded, as pathological findings obtained from surgical operation were defined as the reference standard. However, as discussed above, PSF-PET/CT can provide a considerable impact on diagnosis of lymph node metastasis, which suggests that it could be useful for evaluation before neoadjuvant therapy as well. Thirdly, there was a difference in the iteration numbers between PSF-PET and conventional PET (PSF-PET: 5, conventional PET: 3). However, the PSF-PET iteration number in our study was adjusted to provide a clinically optimal image based on our institutional phantom study, and it has been previously reported that it is usually necessary to increase the number of iterations in order to obtain optimum image when PSF reconstruction is used [13]. In addition, other acquisition parameters including subset and matrix size, which has been reported to affect both the quality and quantitative values of PET images (particularly with PSF reconstruction) [46], were set to the same values for both PSF and conventional PET.

## Conclusions

PSF-PET/CT has potential to provide superior sensitivity for lymph node staging in rectal cancer without reducing specificity compared with conventional PET/CT and pelvic MRI. As PET/CT can provide comparable diagnostic value to MRI for T staging and detect distant metastasis accurately, PSF-PET/CT is a promising methodology for increasing accuracy in staging rectal cancer.

## Abbreviations

AJCC: American Joint Committee on Cancer
DWI: Diffusion-weighted images
EPI: Echo planar imaging
L/B: Lesion-to-background
LN: Lymph node
MTV: Metabolic tumor volume
NEX: Number of excitations
NPV: Negative predictive value
OSEM: Ordered subset expectation maximization
PPV: Positive predictive value
PSF: Point spread function
ROC: Receiver operating curve
ROI: Region of interest
STIR: Short tau inversion recovery
TE: Echo time
TR: Repetition time
